# Implementation of the pharmacovigilance database in the usage of antibiotics in a tertiary care hospital: A pilot study

**DOI:** 10.1002/hsr2.2254

**Published:** 2024-07-17

**Authors:** Karri Juhu Kiran Krushna, Arup Kumar Misra, Pavani Saggurthi, Sushil Sharma, C. Madhavrao, Gaurav Rangari, L. V. Simhachalam Kutikuppala, Golla Varshitha

**Affiliations:** ^1^ Department of Pharmacology All India Institute of Medical Sciences (AIIMS) Mangalagiri Andhra Pradesh India; ^2^ Department of Internal Medicine International School of Medicine (ISM) Bishkek Kyrgyzstan

**Keywords:** adverse drug reactions, antibiotics, antimicrobial resistance, antimicrobial stewardship, pharmacovigilance

## Abstract

**Background:**

Antimicrobial resistance (AMR) has evolved into a severe public health issue that defies all current control strategies and needs multidisciplinary, creative solutions. Antimicrobial stewardship (AMS) activities demand a set of tools and abilities that can be used by health systems. In view of the growing AMR and the difficulty for the surveillance of it in the developing country, this study was conducted to incorporate pharmacovigilance (PV) in the AMS program.

**Materials and Methods:**

A cross‐sectional pilot study was conducted to collect the PV database of antimicrobials induced adverse drug reactions (ADR) from the Adverse Drug Reactions Monitoring Center (AMC) of the institute for a period of 2 months from August 2022 to September 2022. The information from the database was analyzed to estimate the usage of antibiotics from the PV database from AMC and classified it under the Anatomical Therapeutic Chemical, to assess the rationality of the antimicrobial's usage based on “Access,” “Watch,” and “Reserve” (AWaRe) classification, and to assess the ADR of the antibiotics. The analysis was done by using the SPSS version 20.0 for Windows.

**Results:**

The results showed that the prevalence of ADRs were more in adults’ population with preponderance in female. The antibiotics usage was as per with World Health Organization standard and most of the antibiotics used were from the Access group of AWaRe classifications. Tetracyclines and penicillins were the most used antibiotic group of drugs. The number of patients included in the study was 70. Most of the causality assessment was “possible” (62.85%). In the study, almost 90% of the drug was withdrawn and 70% of the patients were in the recovering stage.

**Conclusion:**

Using existing PV approaches to address usage of antibiotics and AMR issues would allow PV to progress as a field, and governments will get a stronger return on their PV investments.

## Highlights

- Antimicrobial resistance (AMR) has evolved into a severe public health issue.
- Incorporating pharmacovigilance (PV) in the antimicrobial stewardship program is essential.
- Using existing PV approaches to address usage of antibiotics and AMR issues would allow PV to progress as a field.

## 1 INTRODUCTION

Antimicrobial resistance (AMR) has evolved into a severe public health issue that defies all current control strategies and needs multidisciplinary, creative solutions.^1^ Antimicrobial stewardship (AMS) activities demand a set of tools and abilities that can be used by health systems.^2^ However, there is a huge capacity gap between the health systems of developed and underdeveloped countries. Developing systems that can conduct laboratory‐based AMR monitoring in a timely and accurate manner will need time and requires long‐term investments for many low‐ and middle‐income countries with limited laboratory resources. Strong global pharmacovigilance (PV) infrastructure may be critical in countries with limited laboratory coverage and capabilities.^3^ In this sense, the AMS framework's PV program can map and quantify antibiotic consumption and AMR burden in areas with low laboratory coverage and capacity. The science of actions related to the detection, assessment, understanding, and prevention of adverse effects or other drug‐related problems is known as PV.^4^ Recent study has highlighted PV's potential importance in preventing the spread of AMR, highlighting the methodology and resources that PV may provide to programs that monitor suspected resistance or antibiotic abuse.^5^, ^6^

In addition to existing AMR monitoring systems, PV databases can provide information on suspected resistance and inappropriate usage. The World Health Organization's (WHO) worldwide action plan, if executed successfully, will considerably reduce AMR and aid in the evaluation of treatment options. The WHO's Essential Medicines List separates antibiotics into three categories based on indication, availability, and awareness: “Access,” “Watch,” and “Reserve” (AWaRe).^7^ If the AWaRe tool is applied according to national criteria, AMR will be reduced, and access will be ensured. When analyzing antibiotic‐targeting efforts, the AWaRe classification is a crucial tool to examine.

These PV data may reveal use‐related issues, allowing prescribers to have a fuller picture during each session. Off‐label usage or drug use in contraindicated indications reports can be shared with bodies in charge of national antimicrobial policies so that decision‐makers have up‐to‐date information. Antibiotics on the Reserve and Watch lists were also discovered in the PV data, which is particularly important in terms of AMR.^8^ Analyzing these data may reveal how and why specific Watch and Reserve antibiotics are possibly abused or taken off‐label, depending on the indications and accessible formulations. An increase in reports could mean that something about the drug has changed. By analyzing a reported increase, it is possible to determine if there have been any changes in the way a medicine is used.^9^ In this article, the PV database from the Adverse Drug Reactions Monitoring Center (AMC) was analyzed for antimicrobial use, rational antimicrobial use as defined by the AWaRe classification, and side effect profiles that may represent the AMR status.

## 2 MATERIALS AND METHODS

### 2.1 Study design

The study was a descriptive cross‐sectional study where the information's were collected prospectively. It was conducted at the AMC at an Institute of National Importance for a duration of 2 months from August 2022 to September 2022.

### 2.2 Sample size

As this was a pilot study, formal sample size was not calculated. Keeping in mind the availability of adverse drug reactions (ADR) with antibiotics, all the suspected ADR form were screened and evaluated. We evaluated 70 ADRs with antibiotics as the suspected drug during the study period.

### 2.3 Inclusion and exclusion criteria

The inclusion criteria for the study were to include all the ADRs with antibiotics as the suspected drug will be screened and analyzed and the exclusion criteria was to exclude all the ADRs occurred by drugs other than antibiotics.

### 2.4 Ethical consideration

The approval for the study was taken from Institutional Ethics Committee and informed regarding the study to the National Coordination Center—Pharmacovigilance Program of India (NCC‐PvPI). The confidentiality of the ADR was maintained by the investigator.

### 2.5 Study procedure

As a routine practice of PV, the patients and healthcare professionals report ADRs directly to the PV Associate of the AMC of the institute. These reports were stored in the computer, the reports were then analyzed and uploaded in the Vigiflow to forward it to the NCC, Ghaziabad. After obtaining ethics clearance, the ADRs form in the PV database of AMC and VigiBase was screened for the proposed study. All the ADRs form with antibiotics as the suspected drug were screened prospectively as well as retro prospectively for the study. The ADRs only connected to antibiotics in hospitalized as well as patients visiting outpatient department (OPD) were examined. All antibiotics, regardless of whether they were given at the same time or at a different interval, were examined for the possibility of ADRs and included in the analysis. Cases of ADRs that might have been brought on by medications taken concurrently, outside antibiotics, were not included in the analysis. We classified the antibiotics as per the AWaRe (Access, Watch, and Reserve) classification to know their rationality.

### 2.6 Statistical analysis

The data was also be screened for its ADRs and its causality assessment. The statistical analysis was done by using the SPSS version 20.0 for Windows. Frequencies with percentages was calculated for nominal and ordinal variables.

## 3 RESULTS

A total of 70 antibiotics induced ADRs were reported to the AMC until between August 2022 to September 2022. In the age group of 19–65 years (*n* = 63, 90%), majority of ADRs occurred with a female preponderance (*n* = 43, 61.42%) (Table 1). Monotherapy constitutes only 56 (80%) drug reactions, while 14 (20%) reactions were from those who received these drugs as a part of a multidrug regimen. In the total 70 antimicrobial‐induced ADRs, 78 antibiotics agents were involved in the ADRs. In the study, antibiotics had been used for various infectious diseases involving all the organ systems. The system organ class involvement was noted as per Medical Dictionary for Regulatory Activities (MedDRA) terminology using version 22.1. The observations revealed that that skin as an organ was involved in almost 78.57% of the total ADRs reported followed by gastrointestinal (*n* = 8, 11.42%); respiratory (*n* = 2, 2.85%) and other systems of renal, nervous, musculoskeletal, etc. (*n* = 5, 7.14%).

**Table 1:** Demographic profile of the reported adverse drug reactions.

Demographic profile *n* (%)
Sex:
Male = 27 (38.57)
Female = 43 (61.42)
Age group:
0–18 = 2 (2.85)
18–60 = 63 (90)
≥60 = 5 (7.14)
Number of adverse drug reactions reported in AMC = 117
Number of adverse drug reactions including only antimicrobials = 70 (59.82)
Number of antimicrobials suspected for the adverse drug reactions = 78
Causality assessment (as per WHO)
Possible = 44 (62.85%)
Probable = 17 (24.28%)
Certain = 9 (12.85%)
Outcome of the adverse drug reactions
Recovering = 49 (70%)
Recovered = 21 (30%)
Action taken after adverse drug reactions
Drug withdrawn = 64 (91.42%)
Drug not withdrawn = 6 (8.57%)

*Note*: *n* = number, % = percentage

Abbreviations: AMC, Adverse Drug Reactions Monitoring Center; WHO, World Health Organization.

Doxycycline (*n* = 17), Amoxicillin/Clavulanic Acid (*n* = 10), Metronidazole (*n* = 8), Ofloxacin/Ornidazole (*n* = 8), and Ciprofloxacin (*n* = 7) were the most common drug involved in ADRs in the study population as per Anatomical Therapeutic Chemical classification (Table 2). In the study, the antibiotics used as per the AWaRe classifications were mostly from the Access group of antimicrobial drugs (43, 59.72%) followed by drugs belonging to Watch group (20, 25.64%). In the study, it was found that 11.53% of antibiotics combinations were not recommended by the WHO whereas 7.69% of the antibiotics were not listed or classified as per AWaRe classifications (Table 3). In the Chi Square test, the association between sex and the groups of AwaRe classification was not significant. Most of the reactions were related “possible” (62.85%) to drugs as determined by causality assessment followed by “probable” (24.28%) and “certain” (12.85%). Most of the patients were recovering (70%) from the ADRs at the time of reporting to the AMC. Nearly 90% of the antibiotics were withdrawn from the treatment when the patient developed ADRs to the suspected antibiotics (Table 4).

**Table 2:** Usage of antimicrobials from the pharmacovigilance database from AMC as per Anatomical Therapeutic Chemical (ATC) classification.

ATC code	Pharmacological subgroup	Antibiotics used
J01A	Tetracyclines	Doxycycline (*n* = 17)
J01C	β‐Lactam antibacterials—Penicillins	Amoxicillin/Clavulanic acid (*n* = 10)
J01D	Other β‐Lactam antibacterials	
	Cephalosporins	Cefixime (*n* = 5)
		Ceftriaxone (*n* = 1)
		Cefuroxime (*n* = 2)
J01F	Macrolides	Azithromycin (*n* = 1)
	Lincosamides	Clindamycin (*n* = 3)
		Lincomycin (*n* = 1)
J01M	Fluoroquinolones	Ciprofloxacin (*n* = 7)
		Norfloxacin (*n* = 1)
		Ofloxacin (*n* = 2)
J01R	Combination of antibacterials:	
	Penicillins with other antibacterials	Piperacillin/Tazobactam (*n* = 3)
	Fluoroquinolones and Ornidazole	Ofloxacin/Ornidazole (*n* = 8)
		Ciprofloxacin/Tinidazole (*n* = 1)
J01X	Other antibacterials:	
	Glycopeptide antibacterial	Vancomycin (*n* = 1)
	Imidazole derivatives	Metronidazole (*n* = 8)
		Ornidazole (*n* = 2)
	Nitrofuran derivatives	Nitrofurantoin (*n* = 5)

*Note*: *n* = number.

**Table 3:** Antimicrobial's usage on the basis of AWaRe classification.

AWaRe categories (number of reports, %)	Antibiotics in the AWaRe categories (number of reports, %)
Access (43, 59.72%)	Doxycycline (17, 21.79%)
	Amoxicillin/Clavulanic Acid (10, 12.82%)
	Clindamycin (3, 3.84%)
	Metronidazole (8, 10.25%)
	Nitrofurantoin (5, 6.41%)
Watch (20, 25.64%)	Cefixime (5, 6.41%)
	Ceftriaxone (1, 1.28%)
	Cefuroxime (2, 2.56%)
	Azithromycin (1, 1.28%)
	Ciprofloxacin (7, 8.97%)
	Piperacillin/Tazobactam (3, 3.84%)
	Vancomycin (1, 1.28%)
Reserve	None
Other or not classified (6, 7.69%)	Lincomycin (1, 1.28%)
	Norfloxacin (1, 1.28%)
	Ofloxacin (2, 2.56%)
	Ornidazole (2, 2.56%)
Not recommended (9, 11.53%)	Ofloxacin/Ornidazole (8, 10.25%)
	Ciprofloxacin/Tinidazole (1, 1.28%)

*Note*: *n* = number, % = percentage, NR = combinations not recommended by World Health Organization (WHO).

Abbreviation: AWaRe, “Access,” “Watch,” and “Reserve.”

**Table 4:** Causality assessment, outcome and action taken for the antimicrobials induced adverse drug reactions.

Antibiotics in the AWaRe categories (number of ADRs, %)	Causality assessment			Outcome		Action taken	
	Possible	Probable	Certain	Recovering	Recovered	W	NW
Doxycycline (17, 21.79%)	11	3	3	13	4	14	3
Amoxicillin/Clavulanic acid (10, 12.82%)	6	4	0	6	4	10	0
Clindamycin (3, 3.84%)	3	0	0	3	0	3	0
Metronidazole (8, 10.25%)	5	1	2	7	1	6	2
Nitrofurantoin (5, 6.41%)	3	2	0	3	2	4	1
Cefixime (5, 6.41%)	4	1	0	4	1	5	0
Ceftriaxone (1, 1.28%)	1	0	0	0	1	1	0
Cefuroxime (2, 2.56%)	2	0	0	2	0	2	0
Azithromycin (1, 1.28%)	1	0	0	1	0	1	0
Ciprofloxacin (7, 8.97%)	2	4	1	2	5	7	0
Piperacillin/Tazobactam (3, 3.84%)	2	1	0	2	1	3	0
Vancomycin (1, 1.28%)	1	0	0	1	0	1	0
Lincomycin (1, 1.28%)	1	0	0	1	0	1	0
Norfloxacin (1, 1.28%)	1	0	0	1	0	1	0
Ofloxacin (2, 2.56%)	1	0	1	2	0	2	0
Ornidazole (2, 2.56%)	1	0	1	2	0	2	0
Ofloxacin/Ornidazole (8, 10.25%)	7	0	1	8	0	8	0
Ciprofloxacin/Tinidazole (1, 1.28%)	0	1	0	0	1	1	0

*Note*: W = drug withdrawn after reaction, NW = drug not withdrawn after reaction.

Abbreviations: ADR, adverse drug reaction; AWaRe, “Access,” “Watch,” and “Reserve.”

## 4 DISCUSSION

Since their introduction as a therapeutic option for various infectious diseases, antibiotics have demonstrated their efficacy in treating several bacterial infections that were once life‐threatening.^10^, ^11^ Antibiotics are safe, effective, and rarely cause side effects when used properly. But if these drugs are given incorrectly, bacterial resistance could develop. This issue, also known as AMR, is one of the biggest global risks to public health in the twenty‐first century.^11^, ^12^ Antibiotics must be used properly to lower the chance of adverse events and prevent ABR. Another issue with public health that needs the most attention is ADRs, particularly in terms of mortality, morbidity, and healthcare expenditures.^13^ Antibiotics are nonetheless one of the most common drug types that cause ADR.^14^, ^15^ Living reviews of PV are being conducted as the world looks for and implements methods to combat the irrational use of antibiotics and the growing AMR. Data should be viewed as a reliable source of information on patterns in alleged resistance and potential irrational usage of antibiotics, particularly in nations with limited resources and testing facilities.

The majority of ADRs in our study were observed in female patients, and this increased incidence in female patients may be related to hormonal changes that take place at various stages of life and may change the pharmacokinetics profile of the drugs.^16^, ^17^ The majority of ADRs were observed in patients between the ages of 19 and 60, which is consistent with Astolfi et al.^18^ Adult patients predominated among all age groups for the greatest number of antibiotics associated ADRs as per the analysis of the distribution of patients by age. It might be due to changes in pharmacokinetics and pharmacodynamics that occur with ageing, associated co‐morbid disorders, the use of numerous medicines, and infectious diseases. Thus, adult patients were more prone to experience antibiotics associated ADRs. In this study, it was observed that antimicrobial induced ADRs account for 59.82% of all ADRs report in the AMC during the study period which was commensurable to the findings in the study done in India and Ghana.^19^, ^20^

According to reports, antibiotics are one of the main causes of ADRs.^21^ In the study, the most common drugs causing ADRs were Doxycycline (*n* = 17), Amoxicillin/Clavulanic Acid (*n* = 10), Metronidazole (*n* = 8), Ofloxacin/Ornidazole (*n* = 8) and Ciprofloxacin (*n* = 7). Results of the present analysis is in accordance with reports from a similar study.^21^ This study demonstrates that the majority of ADRs were caused by the drugs from the group of tetracyclines, penicillins, quinolones and Imidazole which are similar to several other reports.^22^, ^23^ The “AwaRe” categorization of the WHO creates a standard prescription pattern for antibiotics in healthcare facilities with the aim of enhancing the monitoring system for antibiotic use. As the technique identifies antibiotics for empiric treatment or reserving as the last hope, it must be implemented by nations and hospitals to fight the global battle against AMR.^24^, ^25^ In the study, the drugs used in the Access group was 59.72% which is similar to the target set by WHO for using Access group of antibiotics for 60% of patients.^26^ The most common group of antibiotics used in the Access group were tetracyclines (21.79%), penicillins (12.82%) and imidazoles (10.25%). Among the Watch group antibiotics, the antibiotics used were nearly 25.64% which is slightly higher compared with a recent study of India (24.5%) and reports of worldwide use (24.2%) published in 2021.^19^, ^27^ In this study, cephalosporins was the most commonly used groups among the Watch group which may be alarming as it is considered as one of the factors of extended spectrum beta‐lactamase (ESBL) producing microorganisms.^28^, ^29^ The study also observed that there was no use of drugs from the Reserve group of AWaRe classification. This may indicate a chance to limit use by lowering commercial availability and prescription usage.

Among the reported ADRs, common ones were fixed drug eruption (24.28%), itching (21.42%), rashes (12.85%), swelling (11.42%), and vomiting (8.57%). These findings in the study were quite similar to the studies done by Ding et al., and Priyadharsini et al.^30^, ^31^ Our study is in contrast to studies carried out by Arulappen et al.,^32^ which had exanthem as the most common ADR.^32^ In our study, the most affected organ system by antibiotics associated ADRs is skin followed by gastrointestinal, respiratory, renal, hepatic, neurologic, ophthalmic, musculoskeletal, and others. This finding is parallel with the study done by Ibrahim et al.^33^ whereas contrary to the studies conducted by Shamna et al.,^14^ and Dhar et al.,^34^ which claimed that gastrointestinal system was the most affected organ system.^14^, ^33^, ^34^ In our opinion, this difference could either be due to more frequent use of drugs that have more cutaneous reactions in one study or other groups of drugs causing gastrointestinal symptoms in another.

In this study, only ADRs were taken into account which occurs due to antibiotics. Our study has assessed only the causality of the ADRs thus providing basic information regarding the safety profile of antibiotics. WHO causality assessment scale indicated that 62.85% of the reactions were “possible” which is similar to another study.^35^ This was followed by “probable” (24.28%) and “certain” (12.85%). Most of the causality were “possible” as they might be associated with co‐morbidities and taking polypharmacy at the time of occurrence of ADRs.^36^ A retrospective study showed that occurrence of ADRs was significantly associated with more number of drugs prescribed to the patients as it increases the risk of drug interactions compared with other factors of age and gender.^37^, ^38^ Other reasons could be as most of the cases were from OPDs, and the design of the study was cross‐sectional. The meeting between the physician and the patient at the reporting of ADRs was once. At that time of reporting, the offending antibiotics were withdrawn as per the requirement but the outcome was still recovering. Thus, the patients may require follow‐up and which may change the causality from “possible” to “probable” if the patient fully recovered from the ADRs. This study is contrary to the one carried out in North India, where most cases were “probable.”^39^ Due to medical and ethical concerns, the re‐challenge test was never performed, which is a significant study restriction. As a result, only nine reactions were classified as “certain” in the study.

The analysis of the study showed that the suspected drugs was withdrawn (91.42%) in most of the instances. On the other hand, the dose was changed in others, or the suspected drug was not changed in 8.58% of the other cases due to the risk‐benefit analysis of the individual patients, and in some instances, antibiotics were used based on culture and sensitivity reports. Nearly two‐third of the individuals required treatment to recover from the reactions, with many of them receiving symptomatic treatment and some receiving specialized treatment.

The study had to “several” limitations as this small piece of pilot research. First, there is a risk that some ADRs data, particularly from the first few reports, may have been underreported or insufficiently documented as retrospective ADRs were also included in the study. Second, because it was a cross‐sectional study, it was impossible to track the long‐term effects of ADRs. Third, since the current study was a pilot study from a single AMC, the results should be interpreted cautiously due to the recruitment of the limited number of patients when compared to the prevalence of ADRs caused by antibiotics across the nation.

## 5 CONCLUSIONS

Even though this study is a pilot, antibiotic use was extremely widespread and included a wide variety of medications. ADRs linked to antibiotics were prevalent in patients who were adults. To encourage reasonable use, stringent adherence to the hospital's antibiotic guidelines should be implemented. For the purpose of ensuring the safety of medicines, the health system should encourage the spontaneous reporting of ADRs to antibiotics and other medications, adequate recording, and periodic reporting to regional PV centers. The accurate burden of ADRs in terms of patient morbidity and mortality, human resources, and financial resources can be determined with the aid of periodic analysis of antibiotic safety data. This analysis will also assist in developing guidelines and policies to prevent or lessen the frequency and severity of ADRs and contribute to antibiotic stewardship to reduce AMRs in the country.

A few recommendations include the following: early discovery, prompt action, avoiding agents with overlapping toxicity, changing the offending agent and replacing it with an alternate agent. Medical, surgical, clinical pharmacologists, and other healthcare practitioners involved in patient care must all be participating in the PV program to improve the quality of data generated by exchanging and keeping one another informed with useful information. Audio‐visual lectures should be the means for communication to sensitize and raise knowledge among healthcare professionals as well as residents, interns, and nurses for improvements in knowledge and encouraging spontaneous reporting of antibiotics associated ADRs. Additionally, the general public may play a significant role in strengthening ADR detection and reporting at the grass‐roots level if they are well‐informed in their own language about proper use and safety of drugs through various channels, such as television, radio, print media, social media, or public awareness lectures, etc.

## AUTHOR CONTRIBUTIONS

**Karri Juhu Kiran Krushna**: Conceptualization; methodology; writing—original draft; writing—review & editing; data curation; funding acquisition. **Arup Kumar Misra**: Conceptualization; methodology; data curation; writing—original draft; writing—review & editing. **Pavani Saggurthi**: Conceptualization; writing—original draft; writing—review & editing; data curation. **Sushil Sharma**: Conceptualization; methodology; writing—original draft; writing—review & editing; supervision. **C. Madhavrao**: Conceptualization; writing—original draft; writing—review & editing; data curation. **Gaurav Rangari**: Conceptualization; writing—review & editing; writing—original draft. **L. V. Simhachalam Kutikuppala**: Conceptualization; writing—original draft; writing—review & editing. **Golla Varshitha**: Writing—original draft; writing—review & editing; conceptualization.

## CONFLICT OF INTEREST STATEMENT

The authors declare no conflicts of interest.

## ETHICS STATEMENT

Ethical Committee Approval was taken before conducting the study with IEC no. AIIMS/MG//IEC/2022‐23/183 dated 29‐07‐2022 from All India Institute of Medical Sciences (AIIMS), Mangalagiri, Andhra Pradesh, India.

## TRANSPARENCY STATEMENT

The lead author Golla Varshitha affirms that this manuscript is an honest, accurate, and transparent account of the study being reported; that no important aspects of the study have been omitted; and that any discrepancies from the study as planned (and, if relevant, registered) have been explained.

## Data Availability

The data that support the findings of this study are available from the corresponding author upon reasonable request. The data that support the findings of this study are available on request from the corresponding author. All authors have read and approved the final version of the manuscript and Golla Varshitha had full access to all of the data in this study and takes complete responsibility for the integrity of the data and the accuracy of the data analysis.
